# Fumigation Activity against Phosphine-Resistant *Tribolium castaneum* (Coleoptera: Tenebrionidae) Using Carbonyl Sulfide

**DOI:** 10.3390/insects11110750

**Published:** 2020-10-31

**Authors:** Han Kyung Lee, Geunho Jeong, Hyun Kyung Kim, Bong-Su Kim, Jeong-Oh Yang, Hyun-Na Koo, Gil-Hah Kim

**Affiliations:** 1 Department of Plant Medicine, College of Agriculture, Life and Environment Science, Chungbuk National University, Cheongju 28644, Korea; dhthrms1@naver.com (H.K.L.); hoo043@naver.com (G.J.); nshk0917@gmail.com (H.K.K.); hyunnakoo@hanmail.net (H.-N.K.); 2 Plant Quarantine Technology Center, Animal and Plant Quarantine Agency, Gyeongsangbuk-do 39660, Korea; bskim79@korea.kr (B.-S.K.); joyang12@korea.kr (J.-O.Y.)

**Keywords:** *Tribolium castaneum*, phosphine resistance, *Oryzaephilus surinamensis*, phosphine, carbonyl sulfide, fumigation

## Abstract

**Simple Summary:**

Phosphine is one of the most widely used fumigants for the control of stored grain pests in quarantine. However, PH_3_ resistance to many stored pests has been reported. In this study, the fumigation activity of carbonyl sulfide was researched as an alternative fumigant to control PH_3_-resistant pests. In PH_3_ treatment, there was a clear difference in the fumigation activity of domestic strain *Tribolium castaneum* and resistance strain *T*. *castaneum*, but both the d- and r-strains of *T*. *castaneum* showed similar results in the carbonyl sulfide (COS) treatment. Furthermore, both PH_3_ and COS showed no significant difference in fumigation activity in *Oryzaephilus surinamensis*.

**Abstract:**

Phosphine resistance is occurring among stored-grain pests worldwide. This study investigated the fumigation activity of phosphine (PH_3_) and carbonyl sulfide (COS) against domestic strain (d-strain) *Tribolium castaneum*, resistance strain (r-strain) *T*. *castaneum* and *Oryzaephilus surinamensis*. All developmental stages of the pests were exposed to two fumigants (PH_3_ and COS), and the fumigation activity according to the dose and exposure time was evaluated in a 12-L desiccator and 0.5 m^3^ fumigation chamber. The rice sorption rate and quality following exposure to thetwofumigants were evaluated. The mortality was 2.9% in r-strain *T*. *castaneum*, 49.5% in d-strain *T*. *castaneum* and 99.2% in *O*. *surinamensis* when 2 mg/L PH_3_ was used in a 12-L desiccator for 4 h. However, all pest developmental stages showed 100% mortality after 24 h of exposure in the 0.5 m^3^ fumigation chamber, except for the r-strain *T*. *castaneum*. A mortalityof 100% was observed in all of the r-strain *T*. *castaneum* developmental stages at an exposure time of 192 h. For COS applied at 40.23 mg/L and 50 g/m^3^ in the 12-L desiccator and the 0.5 m^3^ fumigation chamber, respectively, 100% mortality was observed across all developmental stages regardless of species and strain. The sorption of COS was 10% higher than that of PH_3_, but there was no significant difference in rice quality compared to that in the control. Therefore, this study suggests that COS can be used for controlling *T*. *castaneum* resistant to PH_3_.

## 1. Introduction

Various methods are used in quarantine to prevent the inflow of foreign pests due to increased trade among countries. Fumigation, heat treatment, low-temperature treatment and ionization energy have been researched and used for pest control during quarantine [1,2,3,4,5]. Of these methods, fumigation is widely used in quarantine, especially against stored-product pests, because it is an effective and economical method [6,7]. Methyl bromide (MB) and phosphine (PH_3_) are widely used in pest control for grains and stored foods, but the availability of MB is limited because it is an ozone-depleting substance, and thus PH_3_ has been used to control pests in quarantine as an alternative [8,9]. However, PH_3_ resistance has been reported in *Rhyzopertha dominica*, *Tribolium castaneum*, and *Sitophilus zeamais*, which requires a long treatment time [10,11,12]. Therefore, due to the increase in PH_3_-resistant pests, an alternative control agent or control method is necessary. Carbonyl sulfide (COS) was developed in Australia as a fumigant to replace MB and PH_3_ and was patented and registered as a grain fumigant in 1993 [13,14,15,16]. COS has been studied in regard to its efficacy in controlling *Amyelois transitella*, *Oryzaephilus surinamensis*, *Carpophilus hemipterus*, *Lasioderma serricorne*,and *Tribolium confusum* as a substitute for MB and PH_3_ to control stored-grain pests [8,17,18,19].

The red flour beetle, *Tribolium castaneum* (Coleoptera: Tenebrionidae), is one of the most important insect pests found in stored foods and grains worldwide [20,21], causing food deterioration and contamination through the production of debris and feces [22,23]. The sawtoothed grain beetle, *Oryzaephilus surinamensis* (Coleoptera: Silvanidae), is also one of the most common pests affecting stored-food products worldwide, causing problems associated with mechanically damaged grains during harvesting and drying [24,25]. In particular, *O. surinamensis* are pests that cause the most serious damage to stored grains and flour [24,26].

*T*. *castaneum* and *O. surinamensis* are important grain pests, damaging 10–40% of stored crops [27]. Recently, a resistant strain to PH_3_ has appeared in various stored pests, and many studies on the diagnosis and characterization of PH_3_-resistant pests have been conducted, with *Tribolium castaneum* and *Oryzaephilus surinamensis* among the investigated pests [28,29,30,31,32,33,34]. Korea is one of the world’s top five grain importers, accounting for 90% of wheat and corn imports [35]. Therefore, even if the PH_3_-resistant pest populations have not yet been found in Korea, there is a high possibility of an influx of stored-product PH_3_-resistant pests from abroad.

This study investigated the effective control of d- and r-strain *T*. *castaneum* and *O*. *surinamensis*, which are pests of stored grains, by evaluating the effects of PH_3_ and COS fumigation. Therefore, the objective of this study was to provide basic data regarding the use of fumigants to control PH_3_-resistant stored-grain pests.

## 2. Materials and Methods

### 2.1. Insects

Individuals of domestic strain *T. castaneum* (d-strain) and *O. surinamensis* were collected from rice mill buildings in Okcheon and Ochang, Chungbuk Province, Republic of Korea. Individuals of the resistant strain *T. castaneum* (r-strain) were provided by the Plant Quarantine Technology Center, Republic of Korea; this strain originated at Murdoch University, Australia. These stored-grain pests were reared in the laboratory at 26 ± 1 °C and 60–70% relative humidity with a 16:8 h (L:D) photoperiod. Flour (800 g), wheat bran (200 g) and dry yeast (70 g) were mixed to serve as a food source, and the beetles were reared in plastic containers (20 cm W × 7 cm L × 8 cm H).

### 2.2. Fumigants

Phosphine (2% PH_3_ + 98% CO_2_) and carbonyl sulfide (97.5% COS + 2.5% CO_2_) were purchased from Korea Nano Gas Co. (Yeoju, Korea).

### 2.3. Fumigation Experiments

The fumigation activities against all developmental stages of *T*. *castaneum* (d- and r-strain) and *O*. *surinamensis* were investigated using PH_3_ and COS in a 12-L desiccator and 0.5 m^3^ fumigation chamber modified according to Kyung et al. [36].

Twenty individuals of each developmental stage of the 3 kinds of stored-grain pests were placed in a Petri dish (5 cm i.d. × 1 cm) containing 0.05 g of food in a 12-L desiccator. PH_3_ and COS were injected using 100 mL and 500 mL gastight syringes (Hamilton, NV, USA), respectively. The d-, and r-strain *T*. *castaneum* and *O*. *surinamensis* were exposed to PH_3_ at various doses for 4 h, while the r-strain *T*. *castaneum* were exposed to PH_3_ at 2 mg/L for 24 h to 250 h of exposure. The COS treatment was conducted with 24 h of exposure to all stored grain pests. The fumigation treatment was applied at 20 ± 1 °C, and the dishes were incubated at 26 ± 1 °C and 60–70% relative humidity with a 16:8 h (L:D) photoperiod after treatment. The controls were not treated with any fumigants.

Each Petri dish (5 cm) containing each developmental stage of the stored-grain pests was placed in a 0.5 m^3^ fumigation chamber (125 × 50 × 80 cm) at a loading ratio of 50% with rice. The Petri dishes were placed in the top, middle and bottom parts of the chamber, respectively. The PH_3_ and COS treatments were administered at 2 g/m^3^ and 50 g/m^3^, respectively, with 24 h of exposure, except in the case of r-strain *T*. *castaneum*, which was exposed to PH_3_ for 192 h.

All experiments were repeated 3 times, and mortality was evaluated 13 days after treatment for larvae, pupae and adults and 20 days after treatment for the egg stage.

### 2.4. Gas Concentration and Sorption Measurements

The gas concentration in each fumigation chamber was measured with a Tedlar gas sampling bag (1 L, SKC, Dorset, UK) using a gastight syringe (100 µL, Hamilton, NV, USA). The collected gas concentration was analyzed with gas chromatography (GC) (Agilent Technology 6890N, and 7890A, Santa Clara, CA, USA). The detector used for PH_3_ was a nitrogen phosphorus detector (NPD), and that used for COS was a flame photometric detector (FPD). The GC conditions were as follows: The GC NPD injector temperature was 250 °C, the oven temperature was 240 °C, the detector temperature was 320 °C, and the column was an HP-5 (0.53 mm × 15 m, Agilent Technology, Santa Clara, CA, USA) operating in splitless mode. The FPD had an injector temperature of 200 °C, detector temperature of 200 °C and oven temperature of 200 °C, and the column was a DB-Sulfur SCD (0.53 mm × 70 m, Agilent Technology, Santa Clara, CA, USA).

The concentration and time (CT) values were determined by collecting gases at 30 min and 1, 4, 8, 12, and 24 h after treatment with PH_3_ and COS, and gas was collected until 48, 96, and 192 h had passed for PH_3_ in 0.5 m^3^ fumigation chamber (AFHB/ACIA, 1989).

The sorption rates of PH_3_ and COS were determined using a 12-L desiccator with 0% and 50% loading ratios (*w/v*) with rice. PH_3_ and COS were applied at doses of 2 mg/L and 50 mg/L, respectively, at 20 °C for 24 h. The gas concentration for sorption was determined at 1, 4, 8, 12, 18 and 24 h after treatment and analyzed following the GC conditions described above. *C*/*C*_0_ (%) values were calculated as the concentration at each time after treatment (*C*) divided by the initial concentration 30 min after treatment (*C*_0_) and multiplied by 100. A 12-L desiccator without rice was used as the control.

### 2.5. Rice Quality Evaluation

The rice quality was evaluated to determine the effects of the two fumigants on rice. Either 2 g/m^3^ PH_3_ or 50 g/m^3^ COS was applied in the 0.5 m^3^ fumigation chamber at a loading ratio of 50% with rice for 48 h of exposure, and then rice quality was measured 24 h after treatment. The control was not treated with any fumigants.

Rice quality was analyzed by collecting 1 kg of rice from each treatment. As a component analysis, the protein and amylose content in 300 g of rice were measured using an Infratec^TM^1241 grain analyzer (Foss, Hilleroed, Denmark). Measurement of the physical change ratio (%) was carried out with 1000 grains of head rice, broken rice, chalky kernels, and damaged rice using a Single-Grain Rice Inspector, model RN-600 (Kett Electric Laboratory, Tokyo, Japan). All measurements were conducted at Chungcheongbuk-do Agricultural Research and Extension Services (Cheongju, Korea).

### 2.6. Statistical Analysis

The fumigation activities of PH_3_ and COS against *T*. *castaneum* (d-strain and r-strain) and *O*. *surinamensis* were analyzed statistically according to the doses and locations, and the exposure times of the r-strain *T*. *castaneum* were also compared using Tukey’s test [37]. The differences in rice quality between the fumigant-treated and untreated rice were analyzed using a *t*-test [37].

## 3. Results

### 3.1. Fumigation Activities of PH_3_ and COS in a 12-LDesiccator

**Figure 1:**
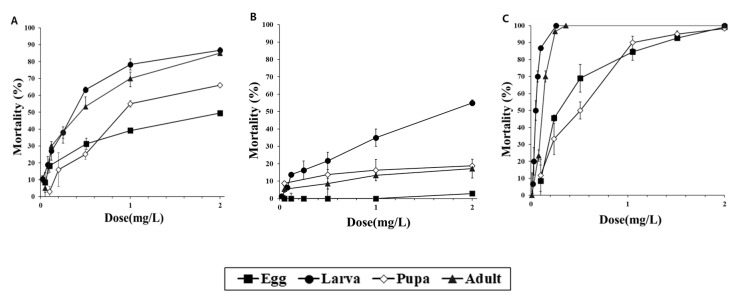
Mortality of (**A**), d-strain *Tribolium castaneum*, (**B**), r-strain *T*. *castaneum* and (**C**), *Oryzaephilus surinamensis* eggs, larvae, pupae and adults after 4 h of exposure to the PH_3_ fumigant in a 12-L desiccator.

**Figure 2:**
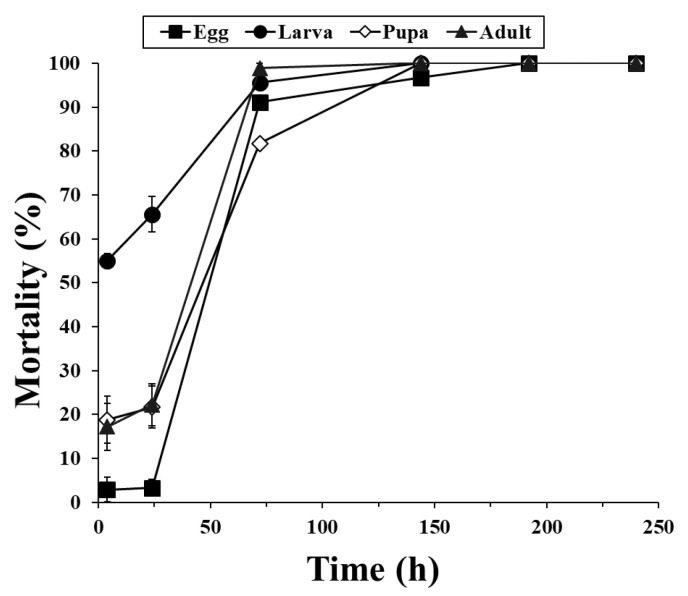
Mortality of r-strain *Tribolium castaneum* across all developmental stages after treatment with 2 mg/L PH_3_ in a 12-L desiccator according to exposure time.

The fumigation activities were investigated after the exposure of d-strain *T*. *castaneum*, r-strain *T*. *castaneum* and *O*. *surinamensis* to PH_3_ for 4 h (Figure 1). Exposure for 4 h was not sufficient to control 100% of d- or r-strain *T*. *castaneum*. Specifically, the r-strain *T. castaneum* showed very low susceptibility across all developmental stages. However, the r-strain did not show 100% mortality after 24 h of exposure (data not shown), but the d-strain *T*. *castaneum* exhibited 100% fumigant activity across all developmental stages under these conditions (Figure 2). In particular, the egg stage showed the lowest mortality of 2.9% after 24 h of exposure to 2 mg/L, and all r-strain *T*. *castaneum* developmental stages showed 100% mortality only when exposed to this amount for 192 h. The egg and pupal stages did not show 100% mortality in the 1 mg/L PH_3_ treatment, but all developmental stages of *O*. *surinamensis* showed >98% mortality (99.2% in egg, 98.3% in pupa, and 100% in larvae and adult stage, respectively) when exposed to 2 mg/L of the PH_3_ treatment for 4 h.

**Figure 3:**
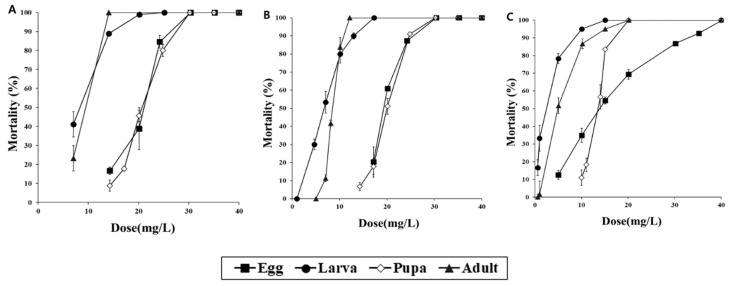
Mortality of (**A**), d-strain *Tribolium castaneum*, (**B**), r-strain *T*. *castaneum* and (**C**), *Oryzaephilus surinamensis* eggs, larvae, pupae and adults after 24 h of exposure to the carbonyl sulfide (COS) fumigant in a 12-L desiccator.

After 24 h of exposure to various doses of fumigation with COS, mortality was evaluated in d-strain *T*. *castaneum*, r-strain *T*. *castaneum* and *O*. *surinamensis* (Figure 3). Treatment with COS had similar results in both d-strain and r-strain *T*. *castaneum*. The d-strain *T*. *castaneum* adults and larvae showed 100% mortalities at 14 mg/L and 25 mg/L, respectively, and the same percentage of activity was observed in r-strain *T*. *castaneum* at 12 mg/L and 17 mg/L. The eggs and pupae of both strains showed 100% mortality only when 30 mg/L of COS was used. Although the fumigation activity detected in COS-treated eggs and pupae was lower than that in other developmental stages, fumigant activity was observed regardless of the strain. *O*. *surinamensis* showed 100% mortality in adult, larva and pupa stages at 20 mg/L, but 100% mortality was observed in the egg stage at 40 mg/L.

### 3.2. Scale-Up Fumigation Experiment with PH_3_ and COS in a 0.5 m^3^ Fumigation Chamber

In the 0.5 m^3^ fumigation chamber experiment, when d-strain *T*. *castaneum* and *O*. *surinamensis* were treated with 2 g/m^3^ PH_3_ for 24 h at a 50% loading ratio with rice, all developmental stages showed 100% mortality regardless of the location (Table 1). However, the r-strain *T*. *castaneum* larvae showed less than 70% mortality only following 24-h exposure to PH_3_, and the other developmental stages showed very low fumigation activities (<29%). When the time of exposure to PH_3_ was increased to 192 h, 100% mortality was observed across all developmental stages of r-strain *T*. *castaneum* at all locations.

The fumigation activity of COS was investigated after 24 h of exposure in the 0.5 m^3^ fumigation chamber for d-strain *T*. *castaneum*, r-strain *T*. *castaneum* and *O*. *surinamensis* (Table 2). The d-strain *T*. *castaneum*, r-strain *T*. *castaneum* and *O*. *surinamensis* exposed to 50 g/m^3^ of COS for 24 h showed 100% mortality regardless of location.

### 3.3. Rice Quality and Sorption of the Two Fumigants

Six rice quality experiments under exposure to PH_3_ and COS were conducted in a 12-L desiccator (Table 3). The changes in protein content and amylose, which are components of rice, did not show any statistically significant differences compared to those in the control for both fumigants. The two fumigants did not affect the rice in terms of the four kinds of physical change rates.

**Figure 4:**
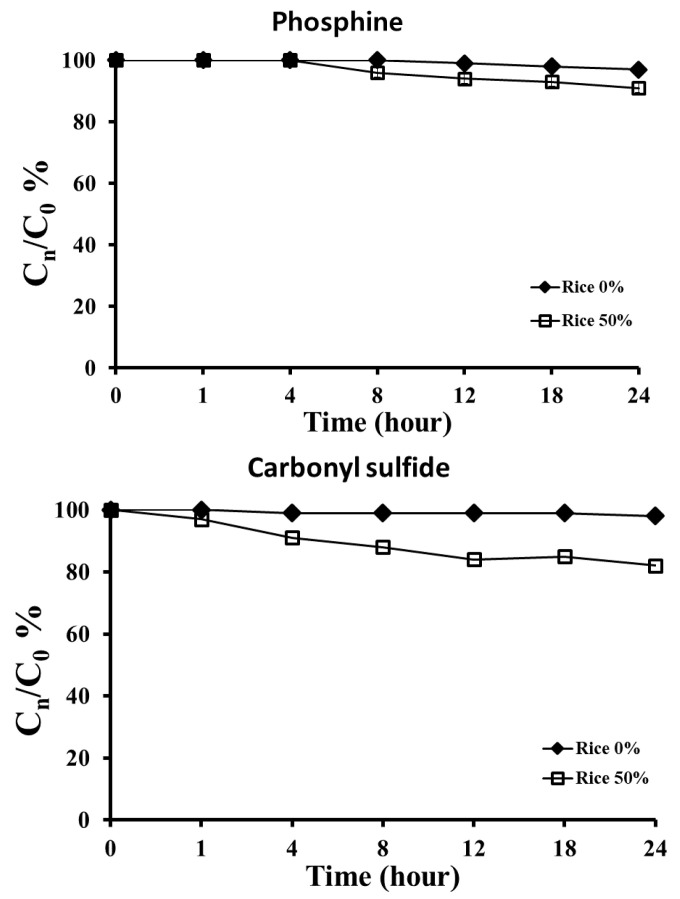
Concentrations of the two fumigants (2 mg/L PH_3_ and 50 mg/L COS) at 20 °C according to the loading ratio (0% and 50%) of rice for 24 h in a 12-L desiccator.

The sorption rates of rice following exposure to PH_3_ and COS for up to 24 h were analyzed (Figure 4). After 5 h of treatment of a 50% loading ratio of rice with 2 mg/L PH_3_, the concentration began to decrease to 96%, and the concentration was 91% after 24 h of treatment. When the 50% loading ratio of rice was treated with 50 mg/L COS, the concentration began to decrease to 97% after 1 h and decreased to 82% after 24 h. However, PH_3_ and COS did not show a significant reduction in concentration (97% and 98%, respectively), even after 24 h of treatment, in the control without rice.

## 4. Discussion

This study evaluated the activity of PH_3_ and COS against two stored-product pests, *T*. *castaneum* (d-strain and r-strain) and *O*. *surinamensis*. PH_3_ shows fumigation activity against various grain pests, *T. castaneum*, *Rhyzopertha dominica*, and *O. surinamensis*, but an increase in the resistant strain of these insects has been reported [11,38,39,40]. This experiment was performed to investigate an alternative fumigant that is effective against PH_3_-resistant pests, especially *T*. *castaneum*. This is because *T. castaneum* has a very high frequency of strong resistance to PH_3_, especially in the United States, Southern India and Turkey [11,29,30]. Although it has been reported that sulfuryl fluoride (SF) has the effect of controlling PH_3_-resistant pests, it is vulnerable to low temperatures and has a disadvantage in that it is not easy to use because there is a change in activity according to sealing techniques during treatment [41]. COS is also toxic to stored-grain pests such as *T. castaneum*, *R. dominica*, *O. surinamensis*, *Callosobruchus chinensis*, *Sitophilus zeamais*, *Lasioderma serricorne*, *Cryptolestes pusillus*, *Callosobruchus maculatus*, *Trogoderma variable*, and *Tribolium confusum* [14,42]. This experiment also showed fumigation activity against *T*. *castaneum* and *O*. *surinamensis.* Ther-strain *T*. *castaneum* treated with PH_3_ showed an increasing fumigation effect only when the exposure time was increased because the fumigation effect was much lower than that observed ford-strain *T*. *castaneum*, but both the d- and r-strains of *T*. *castaneum* showed similar results in the COS treatment. It was also observed that the COS showed higher tolerance to *O*. *surinamensis* than in d- and r-strains of *T*. *castaneum* at the egg stage. This is because of the difference in the target site where PH_3_ and COS exhibit toxicity; PH_3_ is toxic because of the formation of reactive oxygen radicals involved in the electron transport chain, and COS is toxic because of the metabolite action of hydrogen sulfide [43,44,45]. The fumigation activity of both fumigants differed according to the developmental stage exposed. A similar effect has also been observed in association with other pests and fumigants. Under 4 h of exposure, *Frankliniella occidentalis* eggs showed higher tolerance to the fumigation activity of >16 mg·h/L PH_3_ at 5 °C and >84.4 mg·h/L ethyl formate (EF) at 5 °C than other developmental stages [36]. In addition, *Phthorimaea operculella* larvae and eggs showed the highest tolerance during PH_3_ treatment, and *Carposina niponensis* and *Rhynchophorus ferrugineus* showed the highest tolerance in the egg stage [46,47,48]. In this study, a difference in fumigation activity to each developmental stage was also observed, but the fumigation effect decreased in the egg and pupa stage in d- and r-strain *T*. *castaneum* and *O*. *surinamensis* for both fumigants. Even in the scale-up experiment (0.5 m^3^) in which the dose was set based on the results of the 12-L desiccator experiment, the PH_3_ r-strains of *T*. *castaneum* showed 100% mortality for 192-h exposure, but all three kinds of stored pests could be controlled 100% at the same dose in the COS treatment. Thus, even when scaling-up at the field level, it is thought that fumigation activity against pests will be observed at the COS dose (50 g/m^3^) used inthis study.

In terms of the adsorption rate, that of COS was higher than that of PH_3_, but it did not affect the fumigation activity in the 24-h exposure treatment. The use of COS did not have obvious side effects, such as water adsorption by rice, the expansion of cooking rice or dry substances in the rice cooking water, but the flavor changed when a COS concentration of more than 100 g/m^3^ was used [42]. Furthermore, in the rice quality evaluation in this study, both fumigants did not result in any significant difference in quality compared to that ofthe control, even in the treatment of 50 g/m^3^ COS.

These results indicate that PH_3_ is a very effective fumigant for the control of stored-grain pests and that COS could be used an alternative for the control of the *T*. *castaneum* strain resistant to PH_3_ and *O*. *surinamensis*.

## 5. Conclusions

In our evaluation of the fumigation activity of PH_3_ against d- and r-strain *T*. *castaneum* and *O*. *surinamensis*, it was found that r-strain *T*. *castaneum* has very high resistance to PH_3_. However, in the COS treatment, r-strain *T*. *castaneum* showed similar mortality to that of d-strain *T*. *castaneum*. Ifr-strain *T*. *castaneum* appears during the control of stored-grain pests, it is worth considering the use of COS as an alternative to PH_3_.

## Figures and Tables

**Table 1:** Fumigant activity of 2 g/m^3^ PH_3_ at each location in a 0.5 m^3^ fumigation chamber containing rice at a 50% loading ratio.

Exposure Time(h)	Species/Strain	Stages	*n*	Locate	Mortality	CT Value (g·h/m^3^)
	*O. surinamensis*	Egg	283	Top	100 ± 0.0a	39.40
			284	Middle	100 ± 0.0a	
			286	Bottom	100 ± 0.0a	
			285	Control	4.9 ± 2.7b	
		Larva	290	Top	100 ± 0.0a	
			268	Middle	100 ± 0.0a	
			291	Bottom	100 ± 0.0a	
			278	Control	3.6 ± 0.9b	
		Pupa	271	Top	100 ± 0.0a	
			281	Middle	100 ± 0.0a	
			279	Bottom	100 ± 0.0a	
			280	Control	5.8 ± 4.0b	
		Adult	283	Top	100 ± 0.0a	
			291	Middle	100 ± 0.0a	
			290	Bottom	100 ± 0.0a	
			288	Control	5.3 ± 0.8b	
24	*T*. *castaneum*d-strain	Egg	296	Top	100 ± 0.0a	
			296	Middle	100 ± 0.0a	
			293	Bottom	100 ± 0.0a	
			294	Control	9.4 ± 0.8b	
		Larva	270	Top	100 ± 0.0a	
			275	Middle	100 ± 0.0a	
			280	Bottom	100 ± 0.0a	
			270	Control	5.0 ± 1.5b	
		Pupa	270	Top	100 ± 0.0a	
			270	Middle	100 ± 0.0a	
			270	Bottom	100 ± 0.0a	
			270	Control	7.3 ± 1.1b	
		Adult	270	Top	100 ± 0.0a	
			270	Middle	100 ± 0.0a	
			270	Bottom	100 ± 0.0a	
			270	Control	3.3 ± 0.6b	
	*T*. *castaneum*r-strain	Egg	301	Top	3.3 ± 1.9a	
			292	Middle	5.5 ± 1.2a	
			276	Bottom	7.6 ± 1.0a	
			278	Control	4.7 ± 1.4b	
		Larva	279	Top	61.2 ± 2.8a	
			274	Middle	67.1 ± 1.9a	
			278	Bottom	65.8 ± 4.2a	
			281	Control	3.6 ± 1.6b	
		Pupa	297	Top	25.9 ± 1.8a	
			296	Middle	24.0 ± 3.5a	
			291	Bottom	21.7 ± 3.7a	
			291	Control	7.2 ± 1.2b	
		Adult	273	Top	28.3 ± 5.1a	
			284	Middle	27.0 ± 4.3a	
			281	Bottom	23.2 ± 3.9a	
			286	Control	5.3 ± 1.9b	
192	*T*. *castaneum*r-strain	Egg	285	Top	100 ± 0.0a	261.31
			277	Middle	100 ± 0.0a	
			300	Bottom	100 ± 0.0a	
			279	Control	1.8 ± 0.8b	
		Larva	305	Top	100 ± 0.0a	
			285	Middle	100 ± 0.0a	
			281	Bottom	100 ± 0.0a	
			273	Control	7.3 ± 3.1b	
		Pupa	300	Top	100 ± 0.0a	
			300	Middle	100 ± 0.0a	
			300	Bottom	100 ± 0.0a	
			288	Control	7.7 ± 1.3b	
		Adult	291	Top	100 ± 0.0a	
			284	Middle	100 ± 0.0a	
			279	Bottom	100 ± 0.0a	
			280	Control	8.1 ± 2.6b	

Mortality (%, mean ± SE), followed by the different letter within columns, was significantly different at *p* < 0.05 by Tukey’s test (SAS Institute 2009).

**Table 2:** Fumigant activity of 50 g/m^3^ COS at each location in a 0.5 m^3^ fumigation chamber containing rice at a 50% loading ratio.

Exposure Time(h)	Species/Strain	Stages	*n*	Locate	Mortality	CT Value (g·h/m^3^)
24	*O. surinamensis*	Egg	307	Top	100 ± 0.0a	1264.7
			308	Middle	100 ± 0.0a	
			300	Bottom	100 ± 0.0a	
			324	Control	9.7 ± 3.0b	
		Larva	316	Top	100 ± 0.0a	
			301	Middle	100 ± 0.0a	
			328	Bottom	100 ± 0.0a	
			292	Control	7.2 ± 2.6b	
		Pupa	280	Top	100 ± 0.0a	
			320	Middle	100 ± 0.0a	
			319	Bottom	100 ± 0.0a	
			332	Control	10.2 ± 2.5b	
		Adult	317	Top	100 ± 0.0a	
			299	Middle	100 ± 0.0a	
			303	Bottom	100 ± 0.0a	
			285	Control	7.1 ± 2.0b	
	*T*. *castaneum*d-strain	Egg	288	Top	100 ± 0.0a	
			301	Middle	100 ± 0.0a	
			313	Bottom	100 ± 0.0a	
			320	Control	3.0 ± 1.7b	
		Larva	300	Top	100 ± 0.0a	
			300	Middle	100 ± 0.0a	
			300	Bottom	100 ± 0.0a	
			300	Control	4.3 ± 1.2b	
		Pupa	270	Top	100 ± 0.0a	
			270	Middle	100 ± 0.0a	
			270	Bottom	100 ± 0.0a	
			270	Control	8.5 ± 1.6b	
		Adult	282	Top	100 ± 0.0a	
			277	Middle	100 ± 0.0a	
			274	Bottom	100 ± 0.0a	
			275	Control	6.5 ± 2.2b	
	*T*. *castaneum*r-strain	Egg	243	Top	100 ± 0.0a	
			304	Middle	100 ± 0.0a	
			347	Bottom	100 ± 0.0a	
			262	Control	8.4 ± 2.3b	
		Larva	295	Top	100 ± 0.0a	
			307	Middle	100 ± 0.0a	
			272	Bottom	100 ± 0.0a	
			306	Control	5.9 ± 1.7b	
		Pupa	289	Top	100 ± 0.0a	
			285	Middle	100 ± 0.0a	
			288	Bottom	100 ± 0.0a	
			312	Control	9.1 ± 1.4b	
		Adult	343	Top	100 ± 0.0a	
			329	Middle	100 ± 0.0a	
			304	Bottom	100 ± 0.0a	
			278	Control	2.5 ± 1.3b	

Mortality (%, mean ± SE), followed by the different letter within columns, was significantly different at *p* < 0.05 by Tukey’s test (SAS Institute 2009).

**Table 3:** Quality of rice exposed to 2 g/m^3^ PH_3_ and 50 g/m^3^ COS at a 50% loading ratio following 48 h of exposure in a 0.5 m^3^ fumigation chamber.

Quality Criteria ^a^	Control	PH_3_	*p*-Value	COS	*p*-Value
Protein content	5.60 ± 0.00a	5.53 ± 0.03a	0.12	5.60 ± 0.00a	-
Amylose content	13.77 ± 0.0a	14.01 ± 0.06a	0.03	13.80 ± 0.03a	0.35
Head rice ratio	76.80 ± 1.36a	76.0 ± 1.20a	0.68	76.6 ± 1.37a	0.92
Broken rice ratio	1.37 ± 0.23a	1.53 ± 0.35a	0.75	0.90 ± 0.00a	0.32
Chalky rice ratio	7.07 ± 0.38a	7.67 ± 0.37a	0.32	6.67 ± 0.61a	0.91
Damaged rice ratio	14.83 ± 1.18a	12.97 ± 0.92a	0.39	15.8 ± 0.80a	0.52

^a^ A *t*-test was used to compare values (%, mean ± SE) of each rice quality criteria between control and treated fumigants.
